# Lichen Sclerosus in Pediatric Phimosis: Histopathological Prevalence and Lack of Human Papilloma Virus-Related Koilocytosis

**DOI:** 10.7759/cureus.106084

**Published:** 2026-03-29

**Authors:** Jimena Krikorian, Anabella Maiolo, Ignacio Tobia Gonzalez, Sebastian G Tobia González

**Affiliations:** 1 Urology, Sor Maria Ludovica Children's Hospital, La Plata, ARG; 2 Urology, Universidad Nacional de La Plata, La Plata, ARG; 3 Urology, Driscoll Children's Hospital, Corpus Christi, USA

**Keywords:** balanitis xerotica obliterans, circumcision, hpv, lichen sclerosus, pediatric urology, phimosis

## Abstract

Background: Lichen sclerosus (LS), also known as balanitis xerotica obliterans (BXO) when affecting the male genitalia, is a chronic inflammatory dermatosis that can involve the foreskin and glans penis. LS is a chronic inflammatory condition affecting the male genitalia that may lead to progressive preputial fibrosis and pathological phimosis. The etiology of this condition remains unclear, and its relationship with viral infections is still debated, particularly in pediatric populations. The aim of this study was to evaluate the incidence of LS in foreskin specimens obtained during circumcision for phimosis and to assess the presence of koilocytic changes suggestive of human papilloma virus (HPV) infection.

Methods: An observational descriptive study was conducted in pediatric patients undergoing circumcision for phimosis between January 2021 and May 2021 at a tertiary pediatric hospital. Clinical variables included age, type of phimosis according to the Kayaba classification, and clinical suspicion of balanitis xerotica obliterans prior to surgery. All patients underwent circumcision, and foreskin specimens were systematically sent for histopathological examination. The pathological analysis evaluated the presence of LS and koilocytic changes. Statistical analysis included Student’s t-test for continuous variables and chi-square or Fisher’s exact test for categorical variables, considering a p-value lower than 0.05 as statistically significant.

Results: Twenty-six patients were initially included in the study. Two cases were excluded because of incomplete clinical information, leaving a final sample of 24 patients. The mean age was seven years, with a range between 1 and 15 years. All patients presented with Kayaba type I phimosis. Histopathological examination revealed LS in 11 cases (45.8%), while the remaining 13 specimens (54.2%) showed nonspecific chronic inflammatory changes. Clinical suspicion of BXO prior to surgery was documented in seven patients (29.2%), and histopathological confirmation was observed in all of these cases. Additionally, LS was identified histologically in four patients (16.7%) without previous clinical suspicion. This difference between clinical suspicion and histopathological diagnosis was statistically significant (p = 0.01). No cases demonstrated koilocytic changes suggestive of HPV infection.

Conclusions: Lichen sclerosus is a relatively frequent histopathological finding in pediatric patients undergoing circumcision for phimosis, even when clinical suspicion is absent. Routine histopathological examination of foreskin specimens may help identify subclinical disease. In this series, no association was identified between LS and koilocytic changes suggestive of HPV infection. Further studies are necessary to clarify the etiological factors involved in LS and its potential relationship with viral infections.

## Introduction

Lichen sclerosus (LS), also historically referred to as balanitis xerotica obliterans (BXO) when involving the male genitalia, is a chronic inflammatory mucocutaneous disorder characterized by epithelial atrophy, dermal sclerosis, and chronic inflammatory infiltration [1]. The disease may affect the foreskin and glans penis and can lead to progressive scarring and fibrosis of the prepuce, resulting in pathological phimosis and, in more advanced cases, meatal or urethral stenosis.

Although LS is more frequently reported in adults, it is also recognized in pediatric populations and may represent an important underlying cause of pathological phimosis. Histopathological examination of foreskin specimens obtained during circumcision has demonstrated LS in a significant proportion of pediatric patients, suggesting that the condition may be underdiagnosed when relying solely on clinical evaluation [2,3].

The pathogenesis of LS remains incompletely understood. Proposed mechanisms include autoimmune processes, chronic inflammation, genetic predisposition, and infectious factors. Among these, human papillomavirus (HPV) has been proposed as a possible contributor to epithelial alterations observed in LS lesions. Koilocytic changes in epithelial cells have been described as potential markers of HPV infection [4], and HPV DNA has been identified in a subset of LS lesions, suggesting a possible role in epithelial injury or chronic inflammation [5].

However, the relationship between LS and HPV infection remains controversial, particularly in pediatric populations. The aim of this study was to determine the histopathological prevalence of LS in pediatric patients undergoing circumcision for phimosis and to evaluate the presence of koilocytic changes suggestive of HPV infection in this population.

## Materials and methods

An observational descriptive study was conducted at a tertiary pediatric hospital. Pediatric patients undergoing circumcision for the treatment of pathological phimosis between January 2021 and May 2021 were included. The study was conducted following institutional ethical approval, and all data were anonymized prior to analysis. Given the retrospective nature of the study, the requirement for informed consent was waived.

Consecutive pediatric patients meeting the inclusion criteria during the study period were included. Inclusion criteria consisted of pediatric patients undergoing circumcision due to clinically diagnosed phimosis. Patients older than 14 years of age and those with incomplete clinical data were excluded from the analysis.

Clinical variables collected included patient age, type of phimosis according to the Kayaba classification, and the presence of clinical suspicion of lichen sclerosus prior to surgery. The Kayaba classification was used to categorize foreskin retractability, as originally described by Kayaba et al. [6]. Although this classification was used descriptively, surgical indication was based on clinical findings consistent with pathological phimosis, including the presence of a fibrotic preputial ring, recurrent inflammation, or clinical suspicion of lichen sclerosus, rather than classification alone. Clinical suspicion was defined by the presence of a whitish sclerotic ring, induration of the foreskin, or associated meatal involvement.

All procedures were performed using standard circumcision techniques. In every case, the excised foreskin specimen was submitted for routine histopathological examination. Histopathological evaluation was performed by experienced pathologists using standardized diagnostic criteria.

Histopathological examination was performed using hematoxylin and eosin staining. Diagnostic criteria for LS included epithelial atrophy, basal cell degeneration, dermal hyalinization, and a band-like inflammatory infiltrate. Koilocytic changes were defined as perinuclear clearing with nuclear atypia suggestive of HPV-related cytopathic effect.

Continuous variables were summarized as means and ranges. Statistical comparisons were performed using Student’s t-test for continuous variables and chi-square or Fisher’s exact test for categorical variables as appropriate. Statistical significance was defined as a p-value < 0.05. Statistical analysis was performed using IBM Corp. Released 2019. IBM SPSS Statistics for Windows, Version 25. Armonk, NY: IBM Corp., and all tests were two-tailed.

## Results

A total of 26 patients were initially included in the study. Two patients were excluded due to incomplete clinical information, resulting in a final cohort of 24 patients. The mean age of the patients was seven years, with a range between one and fifteen years. All patients presented with type I phimosis according to the Kayaba classification. The clinical and histopathological findings are summarized in Table 1.

**Table 1:** Clinical and Histopathological Findings in Pediatric Patients Undergoing Circumcision for Phimosis Values are presented as numbers (percentages) unless otherwise indicated. LS: lichen sclerosus; BXO: balanitis xerotica obliterans. "Clinical suspicion" refers to a preoperative diagnosis based on physical examination. Histopathological diagnosis was established by microscopic examination of foreskin specimens obtained during circumcision. The chi-square statistic and p-value were calculated to compare clinical suspicion and histopathological diagnosis.

Variable	Value	p-value
Number of patients	24	—
Mean age	7 years (range 1–15)	—
Kayaba type I phimosis	24 (100%)	—
Clinical suspicion of LS	7 (29.2%)	χ² = 6.63, p = 0.01
Histopathological LS	11 (45.8%)	—
Chronic inflammation without LS	13 (54.2%)	—
LS without clinical suspicion	4 (16.7%)	—
Koilocytic changes	0	—

Histopathological examination demonstrated findings consistent with lichen sclerosus in 11 of the 24 foreskin specimens (45.8%). The remaining 13 samples (54.2%) showed nonspecific chronic inflammatory changes without diagnostic features of LS.

Clinical suspicion of balanitis xerotica obliterans prior to surgery was documented in seven patients (29.2%). Histopathological analysis confirmed LS in all seven of these cases. Additionally, four patients without clinical suspicion of LS demonstrated histopathological evidence of LS. The difference between clinical suspicion and histopathological diagnosis was statistically significant (p = 0.01). No specimens demonstrated koilocytic changes suggestive of HPV infection.

## Discussion

Lichen sclerosus represents an important pathological entity in pediatric urology because of its potential association with phimosis and urethral disease. The present study demonstrates that LS was present in nearly half of the foreskin specimens obtained from pediatric patients undergoing circumcision for phimosis. This prevalence is consistent with previously reported rates ranging between 20% and 50% in pediatric circumcision series [2,6-9].

An important observation in this study is the discrepancy between clinical suspicion and histopathological diagnosis. Although all patients with clinically suspected LS were confirmed histologically, additional cases of LS were identified in patients without clinical signs. This finding suggests that LS may remain clinically silent in some pediatric patients and supports the value of histopathological evaluation of foreskin specimens following circumcision.

Lichen sclerosus has also been associated with more extensive urethral disease in some patients. Chronic inflammatory changes and progressive fibrosis may affect the urethral epithelium and contribute to urethral stricture disease in severe cases [7-9]. Early recognition of the disease may therefore be important in preventing long-term complications.

The possible association between LS and HPV infection has been debated for several decades. Some investigators have identified HPV DNA in LS lesions, suggesting a potential viral contribution to epithelial damage and chronic inflammation [5,10-16]. However, other studies have failed to demonstrate a consistent relationship between LS and HPV infection.

In the present series, no cases demonstrated koilocytic changes suggestive of HPV infection. The absence of molecular testing, such as HPV-PCR or in situ hybridization, represents an important limitation of this study, as histopathological evaluation alone may not detect subclinical HPV infection. Therefore, the lack of koilocytic changes should be interpreted with caution, and future studies incorporating molecular diagnostic techniques are warranted. These findings suggest that HPV infection may not play a major role in the pathogenesis of LS in pediatric patients with phimosis; however, no definitive conclusions can be drawn in the absence of molecular confirmation.

The present study has several limitations. The number of patients included was relatively small, which limits the statistical robustness and generalizability of the findings. The single-center design may limit the generalizability of the findings to other populations and clinical settings. The Kayaba classification may have limitations in distinguishing physiological from pathological phimosis and may be subject to interobserver variability. HPV infection was inferred from histopathological findings rather than confirmed by molecular testing. Given the relatively small sample size and the descriptive design of the study, no formal power calculation was performed. As a result, the study may be underpowered to detect less frequent findings, including koilocytic changes, and the possibility of a type II error should be considered. Histopathological interpretation may be subject to interobserver variability, which represents a potential limitation of this study.

The absence of koilocytic changes in all specimens analyzed in this study suggests that HPV may not play a major role in the pathogenesis of LS in pediatric patients; however, these findings should be interpreted with caution, given the lack of molecular confirmation. While HPV DNA has been detected in selected adult cases, pediatric studies have shown inconsistent findings, suggesting that inflammatory and immune-mediated mechanisms may be more relevant in this population.

Further studies, including larger patient populations and molecular diagnostic techniques, may help clarify the potential role of HPV and other infectious agents in the pathogenesis of LS.

## Conclusions

Lichen sclerosus is a frequent histopathological finding in pediatric patients undergoing circumcision for phimosis and may be present even in the absence of clinical suspicion. Routine histopathological evaluation of foreskin specimens may help identify subclinical disease. In this series, no association was found between lichen sclerosus and koilocytic changes suggestive of HPV infection.
